# Phenotype and functionality of follicular helper T cells in patients with acute dengue infection

**DOI:** 10.1186/s12929-020-00641-2

**Published:** 2020-04-08

**Authors:** Ayesha Wijesinghe, Jayani Gamage, Hemantha Goonewardena, Laksiri Gomes, Deshni Jayathilaka, Dulharie T. Wijeratne, Ruklanthi de Alwis, Chandima Jeewandara, Ananda Wijewickrama, Graham S. Ogg, Gathsaurie Neelika Malavige

**Affiliations:** 1 Centre for Dengue Research, University of Sri Jayewardenepura, Nugegoda, Sri Lanka; 2 Programme in Emerging Infectious Diseases, Duke-NUS Medical School, Singapore, Singapore; 3 Viral Research & Experimental Medicine Centre, SingHealth/Duke-NUS, Singapore, Singapore; 4 National Institute of Infectious Diseases, Angoda, Sri Lanka; 5 MRC Human Immunology Unit, Weatherall Institute of Molecular Medicine, Oxford NIHR Biomedical Research Centre, University of Oxford, Oxford, UK

**Keywords:** Follicular helper T cells, Plasmablasts, IL-21, Neutralizing antibodies, NS1 antibodies, Viral loads

## Abstract

**Background:**

The association of functionality and phenotype of follicular helper T cells (Tfh) with dengue virus (DENV) specific antibody responses and clinical disease severity has not been well studied.

**Methods:**

We investigated the phenotype and functionality of Tfh cells and plasmablasts in adult patients (DF = 18, DHF = 22) with acute dengue (day 4 to 8 since onset of fever) of varying severity using multiparametric flowcytometry. The properties of Tfh cells were correlated with viraemia, disease severity, plasmablast responses and DENV-specific serum antibody responses. We further evaluated the kinetics of neutralizing antibodies (Neut50) throughout the course of illness in order to evaluate their association with clinical disease severity and viraemia.

**Results:**

Tfh cells (especially those producing IL-21 and co-expressing PD-1 and ICOS) were found to be significantly expanded (*p* < 0.0001) and highly activated in patients with DHF compared to those with DF. The frequency of Tfh cells significantly correlated with DENV-specific IgG, NS1-specific antibodies and Neut50 antibody titres in patients with DHF but not in those with DF. Although the Neut50 titres increased during the course of acute secondary DENV infection, they showed differences based on serotype. For instance, the Neut50 titres were significantly higher during the latter part of illness in patients with DF compared to DHF in DENV1 infection, while in DENV2, patients with DHF had significantly higher titres. The viral loads during early illness did not correlate with the subsequent rise in the Neut50 antibody titres during any time point of illness.

**Conclusions:**

The expansion of Tfh cells is associated with DHF and DENV-specific IgG, NS1-specific and neutralizing antibodies. Neut50 titres did not associate with disease severity or viraemia at the point of first presentation during the febrile phase, but later titres do show differential association with severity in patients with DENV1 compared to DENV2.

## Background

Dengue viral infections represent one of the most rapidly emerging mosquito borne viral infections in the world, resulting in 390 million infections annually, with an estimated annual global cost of $8.9 billion [1, 2]. The WHO has named dengue as one of the ten threats to global health in 2019 [3]. Intense disease monitoring with meticulous fluid management is currently the only management option, as specific treatments options are not yet available. The only registered dengue vaccine, was shown to have poor efficacy against some DENV serotypes and has a potential to increase the risk of severe disease in dengue seronegative individuals [4–6]. Although infection with a particular serotype of the DENV is thought to result in lifelong immunity, there have been instances where infection with the same serotype occurs despite the presence of neutralizing antibodies specific for that serotype [7, 8]. In addition, some individuals who had high neutralizing antibody titres to DENV2 following vaccination, subsequently developed infection with the same serotype [9]. Therefore, as the correlates of protection against the DENV are still not clear, it is essential to understand the role of T cells and antibodies in the protection against dengue infection.

The role of both conventional CD4+ and CD8+ T cells in dengue has been well studied and show they are highly activated [10–13], and recent investigations have shown that T cells in dengue are likely to have a protective role [10, 12]. However, apart from eliminating cells infected by the DENV and production of antiviral cytokines, certain subsets of T cells such as the Tfh cells also determine the type and magnitude of development of virus specific antibody responses. Tfh cells, which are located in the germinal centre, play a crucial role in activating germinal centre B cells leading to activation, isotype switching and production of long lasting neutralizing antibodies [14, 15]. Some Tfh cells are also found in the peripheral circulation and are identified by the expression of CXCR5. They also upregulate PD-1, ICOS, Bcl-6, CD40 and produce IL-21, which are important for the activation of germinal centre B cells [15]. It was recently shown that Tfh cells are highly activated in acute dengue and also correlated with the frequency of plasmablasts [16]. The Tfh cells were also shown to be able to produce IL-21 and IFNγ upon stimulation with DENV peptides, suggesting that they were capable of providing B cell help, which is largely dependent on production of IL-21 [17]. Therefore, increase in frequency and activation of Tfh cells could lead to higher DENV specific antibody levels which may lead to protection, or possibly disease enhancement [18, 19]. In order to further understand the pathogenesis of acute dengue and the constituents of a protective immune response, it would be important to study how the functionality and phenotype of Tfh cells associate with DENV-specific antibody responses and clinical disease severity.

Dendritic cells (DCs) are crucial in activating naïve CD4+ T cells and inducing their differentiation to different CD4+ subsets [20]. DENV was shown to activate both RIG-I and MDA5 resulting in production of IFNβ and STAT1 phosphorylation, which led to production of IL-27 by both monocyte-derived and skin-derived DCs [21]. This in turn was shown to lead to polarization of naïve CD4+ T cells into Tfh cells by DENV infected DCs [21]. The Tfh cells activated B cells, and indeed a massive plasmablast expansion has been seen in patients with acute dengue [22, 23]. Monocytes, which are important antigen presenting cells are one of the main cell types infected by the DENV during acute illness [24, 25]. Recently we showed that monocytes of healthy individuals with past severe dengue responded to infection with the DENV by producing higher viral loads, increased levels of inflammatory cytokines and altered gene expression compared to monocytes of healthy individuals who had past inapparent dengue infection [26]. Therefore, it is possible that monocytes and DCs of those who are likely to develop severe dengue cause increased activation and differentiation of Tfh cells to produce protective or pathogenic virus specific antibodies.

In this study, we investigated the phenotype and functionality of Tfh cells in acute dengue (acute illness) in patients with varying severity of acute dengue and studied the association of Tfh responses with plasmablast expansion, DENV specific IgM and IgG antibodies, NS1 specific antibodies and DENV serotype specific neutralizing antibody titres to further understand how the functionality and phenotype of Tfh cells associate with expansion of plasmablasts and DENV specific antibody responses in acute dengue infection.

## Methods

### Patients for Tfh cell and plasmablast analysis

Forty adult patients with varying severity of acute dengue infection (during acute illness) were recruited from the National Institute of Infectious Disease, Sri Lanka following written informed consent. The first blood sample was collected during days 4–8 since onset of fever and a convalescent blood sample was obtained on 21–30 days following the onset of fever (when the patient had fully recovered). The patients were assessed several times a day and all clinical features such as the blood pressure, urine output and the presence of bleeding manifestations were recorded. Ultrasound scans were performed to determine the presence of fluid leakage in pleural and peritoneal cavities. Full blood counts were performed several times a day throughout the course of illness. Clinical disease severity was classified according to the 2011 World Health Organization (WHO) dengue diagnostic criteria [27]. Accordingly, patients with ultrasound scan evidence of plasma leakage or having a rise in the haematocrit of ≥20% from the baseline were classified as having DHF. Shock was defined as having cold clammy skin, along with a narrowing of pulse pressure of ≤20 mmHg. Based on the above criteria, 18 patients were classified as having DF and 22 patients were classified as having DHF. Twelve healthy individuals were also recruited as healthy controls for the analysis of the frequency and phenotype of Tfh cells and plasmablasts.

### Patients for investigating the changes in Neut50 titres and viral loads throughout the course of illness

In order to investigate the evolution of the Neut50 titres throughout the course of illness and to determine if it associated with clinical disease severity and the degree of viraemia, we recruited a different cohort of 39 patients with acute secondary DENV1 infection (*n* = 22) and with acute secondary DENV2 infection (*n* = 17). All these patient blood samples were obtained during the acute illness, from the time they were admitted to hospital, until they were discharged. Of those with acute secondary DENV1 infection, 12 patients had DF and 10 patients had DHF. Of those with acute secondary DENV2 infection, 8 patients had DF and 9 patients had DHF (the criteria for classification of patients as secondary dengue is given below). The first blood sample was obtained early during the course of illness (on the day of admission), before any of the patients had gone into the vascular leakage (critical phase). Therefore, by obtaining daily blood samples in this cohort of patients, we could study the changes in the Neut50 titres along with the viral loads, well before patients developed any complications. All clinical and laboratory features were recorded as above and clinical disease severity was classified according to the WHO 2011 guidelines [27].

### Ethics statement

Ethical approval was obtained by the Ethics Review Committee of the Faculty of Medical Sciences, University of Sri Jayewardenepura (Ethics application number: 12.15). All patients were recruited following written informed consent.

### Qualitative and quantitative assessment of viral loads

Dengue infection was confirmed by quantitative real time PCR and the infecting DENV was serotyped and viral loads were quantified as previously described in blood samples obtained during the acute illness [28]. Viral RNA was extracted from serum samples using QIAamp Viral RNA Mini Kit (Qiagen, USA) according to the manufacturer’s protocol. Multiplex quantitative real-time PCR was performed as previously described using the CDC real time PCR assay for detection of the DENV and modified to quantify the DENV [29]. Oligonucleotide primers and a dual labelled probe for DENV 1,2,3,4 serotypes were used (Life technologies, USA) based on published sequences. In order to quantify viruses, standard curves of DENV serotypes were generated as previously described by Fernando, S. *et.al* [28].

### Analysis of DENV specific IgM and IgG levels

Dengue antibody assays were performed using a commercial capture-IgM and IgG Enzyme- Linked Immunosorbent Assay (ELISA) (Panbio, Australia). Based on the WHO criteria, patients with an IgM: IgG ratio of > 1.2 were classified as having a primary dengue infection, while patients with IgM: IgG ratios < 1.2 were categorized under secondary dengue infection [27]. Based on these criteria 13 patients had a primary dengue infection and 27 patients had a secondary dengue infection.

### Flow cytometry for identification of Tfh cells and plasmablasts

Cryopreserved peripheral blood mononuclear cells (PBMC) were thawed in RPMI (Gibco, Life Technologies, USA) at 37 °C. Cells were washed in staining buffer (phosphate buffered saline (PBS) containing 2% FBS) (Gibco, Life Technologies, USA) and stained with Zombie Green (Biolegend, USA) to determine the percentage of dead cells. All the samples that were included in the analysis of Tfh cells and plasmablasts had a cell viability of a median of 77.95% (IQR 72.97 to 88.6%) when stained with the Zombie green stain.

For identification and phenotyping of Tfh cells, surface staining was carried out with monoclonal antibodies: anti-CD3 APC/Cy7 (clone OKT3), anti-CD4 pacific blue (clone OKT4), anti-CXCR5 BV711 (clone J252D4), anti-PD-1 BV605 (clone EH12.2H7) and anti-ICOS APC (clone C398.4A), all purchased from Biolegend (San Diego, California, USA). The cells were then fixed in fixation buffer (Biolegend, USA) and permeabilized in intracellular permeabilization wash buffer (Biolegend, USA), followed by staining with anti-IL-21 PE (clone 3A3-N2) (Biolegend, USA) according to the manufacturer’s instructions. Cells were acquired on a Guava easyCyte 12 and analysed with de-novo FCS Express version 6.

Tfhs were identified by gating of initially CD3+ and CD4+ T cells and then gating on CD4+ T cells which expressed CXCR5. They were further phenotyped based on their expression of ICOS, PD-1 and IL-21. The gating strategy for phenotyping Tfh cells is shown in supplementary figure 1. Fluorescence Minus One (FMO) controls for each antibody were used to determine the gates.

The following antibodies were used for plasmablast staining: anti-CD19 PE/Cy7 (clone HIB19), anti-CD27 pacific blue (clone M-T271), anti-CD38 APC/Cy7 (clone HIT2) and anti-IgD APC (clone IA6–2), all purchased from Biolegend (San Diego, California, USA). The gating strategy for phenotyping plasmablasts is shown in supplementary figure  3. FMO controls were used to determine the gates for each antibody.

### Determining NS1 antibody levels in patient sera

In order to measure NS1 antibody levels in patient serum samples, an in-house indirect ELISA was carried out as previously described by Jayathilaka, D. *et.al* [18]. Serum samples diluted at 1:5000 were added to 96-well plates coated with DENV-2 NS1 protein (Native antigen, USA) and blocked with blocking buffer (PBS containing 0.05% Tween 20 and 1% Bovine serum albumin (BSA). The ELISA was developed with the use of Goat anti-human IgG biotinylated antibody (Mabtech, Sweden), Streptavidin Alkaline Phosphatase enzyme (Abcam, UK) and Para-nitro-phenyl-phosphatase (PNPP) (Thermo Fisher Scientific, USA) substrate. The plate was read on MPSCREEN MR-96A ELISA reader.

### Focus reduction neutralization test (FRNT) for determining neutralizing antibody titres

DENV1 - West Pac 74, DENV2 - S16803 (kindly donated by Prof. Aravinda de Silva) was propagated in C6/36 cell lines and the virus concentration was determined by focus forming assays on Vero-81 cells and expressed as FFU/ml. Using these concentrations, neutralizing antibody titres were assessed by FRNTs. Briefly, Vero-81 cells were seeded overnight on tissue culture treated 96-well plates. An eight-point dilution series of patient serum diluted at 1:5 in DMEM (Gibco, Life Technologies, USA) supplemented with 2% FBS were incubated with a constant amount of DENV1 West Pac 74 and DENV2 S16803 for 1 h at 37 °C with 5% CO_2_. Serum–virus mixtures were then transferred onto the confluent Vero-81 monolayer and incubated for 1 h at 37 °C. Following the addition of Carboxymethyl Cellulose overlay media, the monolayer was incubated at 37 °C with 5% CO_2_ for 2–3 days. Following incubation, the plates were fixed with 4% Paraformaldehyde (Alfa Aesar, UK) and blocked with a blocking buffer (1X perm buffer (Biolegend, USA) containing 3% Normal Goat Serum) (Sigma, USA). Foci were stained using a mix of 4G2 and 2H2 (diluted at 1:1000 in blocking buffer) (kindly donated by Prof. Aravinda de Silva) followed by HRP conjugated goat anti-mouse IgG (diluted at 1:500 in blocking buffer) (KPL, SeraCare Life Sciences, USA) and developed using TrueBlue Peroxidase Substrate (KPL, SeraCare Life Sciences, USA). All assays were done in duplicate. Spots were counted using RStudio software [30] and data were analysed using GraphPad Prism version 7 software. The neutralization percentages were plotted against the log (1/dilution) values, and the Neut50 was calculated with GraphPad Prism 7.

### Statistical analysis

Data analysis was performed using GraphPad Prism version 7 software. As the data were not distributed normally, differences in means were compared using the Mann-Whitney U test (two-tailed). Wilcoxon signed-rank test was used to compare the differences in means of the acute and convalescent samples. The results were expressed as the median and the interquartile range (IQR). To determine positive and negative correlations, the Spearman’s two- tailed correlation test was used. Statistical significance was set at *p* < 0.05.

## Results

Of the 40 patients included in the study to investigate the functionality and phenotype of Tfh cells, the median duration of illness at the time of recruitment was 5 (IQR 4.2 to 6) days. All were found to be infected with the DENV2 serotype, as DENV2 was the only circulating DENV serotype during this period [31]. Their mean age was 35.98 years (SD ± 12.3). Eighteen patients had DF and 22 had DHF and all these patients had a similar duration of illness at the time of recruitment. One of the patients with DHF developed shock. Mild bleeding manifestations such as epistaxis and gum bleeds were seen in five patients. All the samples were obtained from patients with DF and DHF at the same time (median day of illness 5, IQR 4.2 to 6 days). During this epidemic of DENV2 infection, those who subsequently developed DHF, entered the critical phase during day 3 to 4 of illness (median day of entering the critical phase 3) [31]. Therefore, samples were obtained from those with DHF in this study while they were in the critical phase. All patients with DF and DHF were followed throughout the illness and none of the patients with DF later developed fluid leakage and progressed to develop DHF. The clinical and laboratory features of these patients are described in supplementary table 1. According to the results of the commercial capture-IgM and IgG ELISA, 13/40 had primary infection (dengue specific IgM:IgG ratios > 1.2) while the remaining 27 had secondary infection (dengue specific IgM:IgG ratios < 1.2). The definitions of terms with regard to dengue infections and classification of primary and secondary dengue are provided in supplementary table 2.

### Expansion of Tfh cells during acute dengue illness

**Fig. 1:**
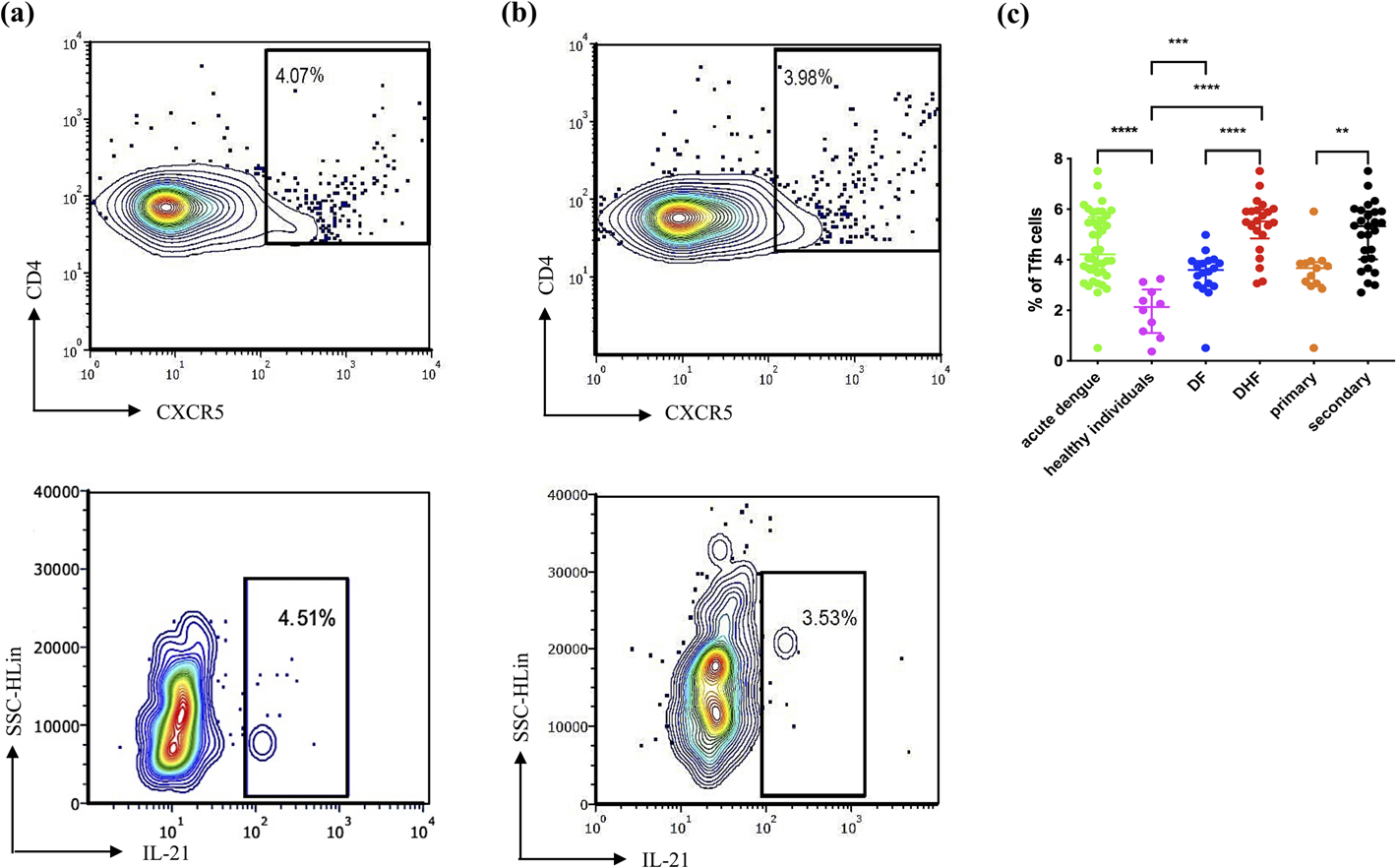
The frequency of Tfh cells and IL-21 producing Tfh cells in patients with acute dengue in the acute phase. The expression of total Tfh cells (cells expressing CD4 + CXCR5+) and IL-21 producing Tfh cells in two patients; patient **a** with acute dengue infection (day 7 since onset of illness) and patient **b** with acute dengue infection (day 6 since onset of illness) are shown. The frequency of Tfh cells (**c**) was assessed in patients with acute DENV2 infection (*n* = 40), in healthy individuals (*n* = 12), in patients with DF (*n* = 18), in patients with DHF (*n* = 22), in patients with primary infection (*n* = 13) and secondary dengue infection (*n* = 27) by multiparametric flowcytometry. The lines display the median and the IQR. **P < 0.01, ***p < 0.001, ****p < 0.0001

As CXCR5 is a key surface molecule expressed on Tfh cells, its expression has been used as an important marker of Tfh cells [17]. Therefore, we identified Tfh cells as CXCR5 expressing CD4+ T cells for identification of peripheral Tfh cells as described previously [17, 32]. As CXCR5 expression was shown to be altered upon stimulation of T cells [17], the Tfh cells were identified *ex vivo* without any stimulation and the intracellular cytokine staining (ICS) for IL-21 was also carried out *ex vivo* without stimulation. We further phenotyped the Tfh cells based on the expression of PD-1, ICOS and IL-21 (Supplementary fig. 1). Representative flow cytometry plots for total Tfh cells and IL-21 producing Tfh cells in two patients with acute DENV infection are shown in Fig. 1a and b, respectively.

The frequency of Tfh cells in patients with acute illness was significantly higher (*p* < 0.0001) when compared to the frequencies in healthy individuals. The frequency of Tfh cells was also significantly higher (*p* < 0.0001) in patients with DHF than in patients with DF. In addition, a significant expansion (*p* = 0.001) was observed in patients with secondary dengue infection compared to those with primary dengue infection (Fig. 1c). Nine patients with DF had a primary infection, while 9 had a secondary dengue infection. There was no difference in the Tfh cell frequency in those with primary or secondary dengue infection, who had DF (*p* = 0.66). Furthermore, 4 patients with DHF had a primary infection, while 18 had a secondary dengue infection. In patients who had DHF, the frequency of Tfh cells was significantly higher (*p* = 0.03) in those with secondary dengue infection compared to those with primary dengue infection. Therefore, these results show that the Tfh cells were significantly expanded in patients with DHF compared to those with DF and healthy individuals, and were also significantly higher in those with secondary dengue infection.

### Phenotypic analysis of Tfh cells in acute dengue

**Fig. 2:**
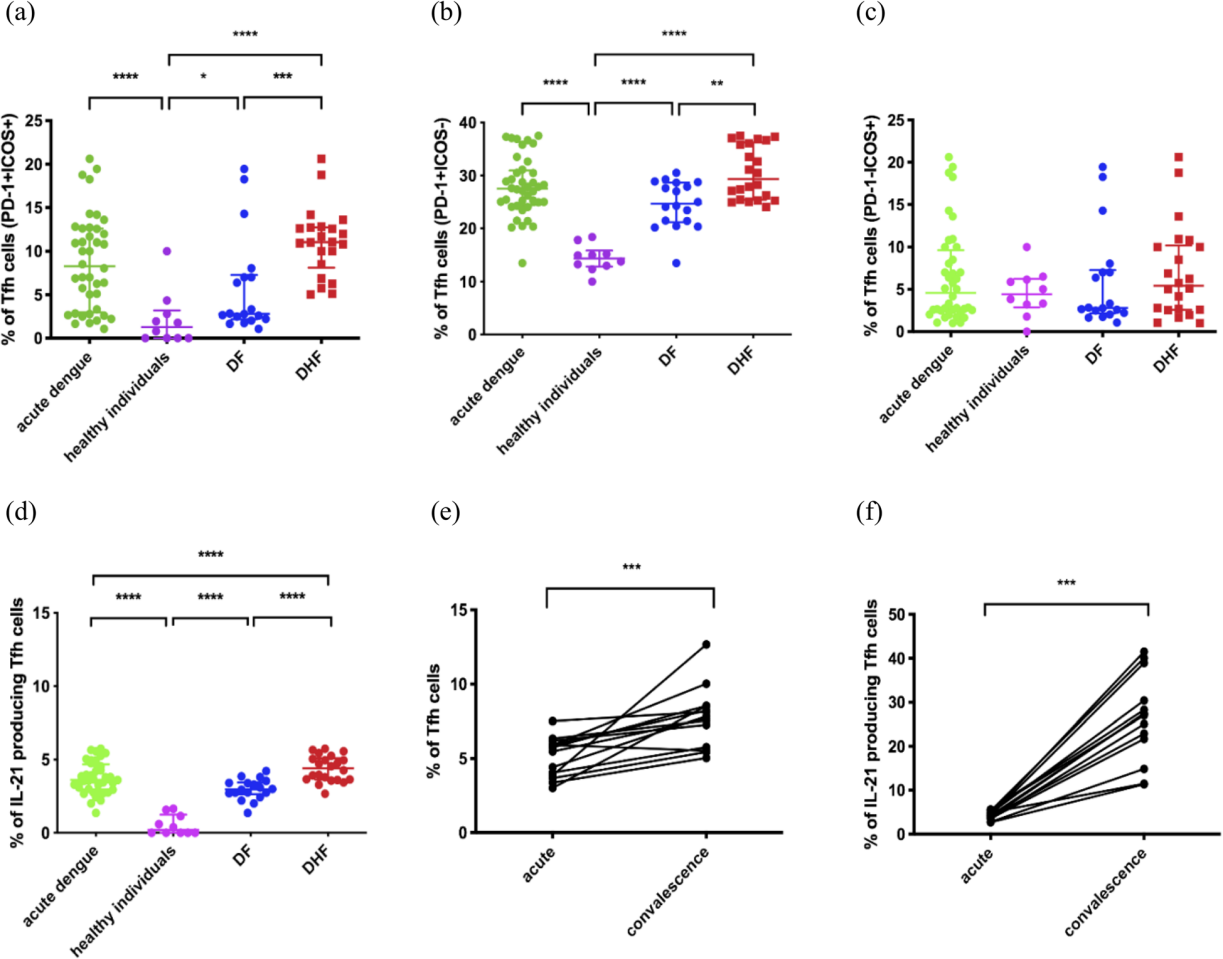
Phenotypical and functional differences of Tfh cells in patients with varying severity of acute dengue. The frequency of PD-1 + ICOS+ co-expressing Tfh cells (**a**), the frequency of Tfh cells expressing PD-1+ alone (**b**), ICOS alone (**c**), and those which produce IL-21 (**d**) were measured in patients with acute DENV2 infection (*n* = 40), in healthy individuals (*n* = 12), in patients with DF (*n* = 18) and DHF (*n* = 22). The frequency of the total Tfh cells (**e**) and IL-21 producing cells (**f**) were compared in a subset of these patients (*n* = 14) during the acute and the convalescent phase of illness. The lines display the median and the IQR. *P < 0.05, **P < 0.01, ***p < 0.001, ****p < 0.0001

PD-1 and ICOS have important functions in the development, functionality and migration of Tfh cells to the germinal centres and are upregulated upon activation [15, 33]. Tfh cells which express both PD-1 and ICOS are shown to be the most efficient helpers of the germinal centre B cells in antibody production [15]. In our study, PD-1 and ICOS co-expressing Tfh cells were significantly expanded (*p* < 0.0001) in patients with acute dengue compared to healthy individuals. PD1 + ICOS+ Tfh cells were also significantly higher (*p* = 0.0009) in patients with DHF compared to patients with DF (Fig. 2a).

The majority of Tfh cells in patients with acute illness only expressed PD-1 and not ICOS. Again, the PD-1 + ICOS- Tfh cells were significantly increased (*p* < 0.0001) in patients with acute dengue infection (Fig. 2b). The frequency of PD-1 + ICOS- Tfh cells was also significantly higher (*p* = 0.001) in patients with DHF compared to those with (Fig. 2b). In contrast, the frequency of PD-1-ICOS+ Tfh cells was not significantly different in acute infection (*p* = 0.70) compared to healthy individuals and in patients with DHF and DF (*p* = 0.43) (Fig. 2c). Therefore, in summary these data show that PD-1 and ICOS co-expressing Tfh cells are significantly expanded in patients with DHF compared to those with milder disease, suggesting that they are actively inducing antibody production by B cells. However, most of the Tfh cells only expressed PD-1 and not ICOS. Recently PD-1 expressing Tfh cells were shown to inhibit the T cell receptor signaling [34]. Therefore, expression of PD-1 alone in the absence of ICOS could be regulating the extent of B cell activating by Tfh cells.

### Functionality of Tfh cells during acute dengue

Tfh cells produce high levels of IL-21, a cytokine that is critical for germinal centre formation and for inducing isotype switching of antibodies produced by germinal centre B cells [14]. Therefore, in order to investigate the cytokine production potential of Tfh cells in acute dengue, we sought to determine IL-21 production by the Tfh cells in patients with acute dengue *ex vivo*. The frequency of IL-21 producing Tfh cells were highly significant (*p* < 0.0001) in patients with acute dengue when compared with healthy individuals, where hardly any cytokine production was observed (Fig. 2d). The frequency of IL-21 producing Tfh cells was also significantly higher (*p* < 0.0001) in patients with DHF compared to those with DF (Fig. 2d).

As the frequencies of Tfh cells were assessed in the acute phase, we proceeded to investigate the phenotype and functionality of these cells in the convalescent period (21 to 30 days since onset of illness) in 14/40 in this cohort. Representative flow cytometry plots for total Tfh cells and IL-21 producing Tfh cells during the convalescent phase are shown in supplementary figure 2a and b, respectively. Interestingly, the frequency of Tfh cells was significantly higher (*p* = 0.0002) during the convalescent phase in these patients compared to the frequencies in acute phase (Fig. 2e). Furthermore, the frequency of IL-21 producing Tfh cells was significantly higher (*p* = 0.0001) during convalescent phase compared to acute phase of illness (Fig. 2f). However, the proportion of Tfh cells positive for PD-1 was significantly less (*p* = 0.0004) in the convalescent phase compared to the acute phase. As the antibody titres have shown to be highest during the convalescent phase/recovery phase of dengue, our data suggest that the Tfh cells continue to activate B cells throughout this period, to induce DENV-specific antibody production.

### Plasmablast expansion in acute dengue infection and correlation of frequency of plasmablasts with frequency of Tfh cells

**Fig. 3:**
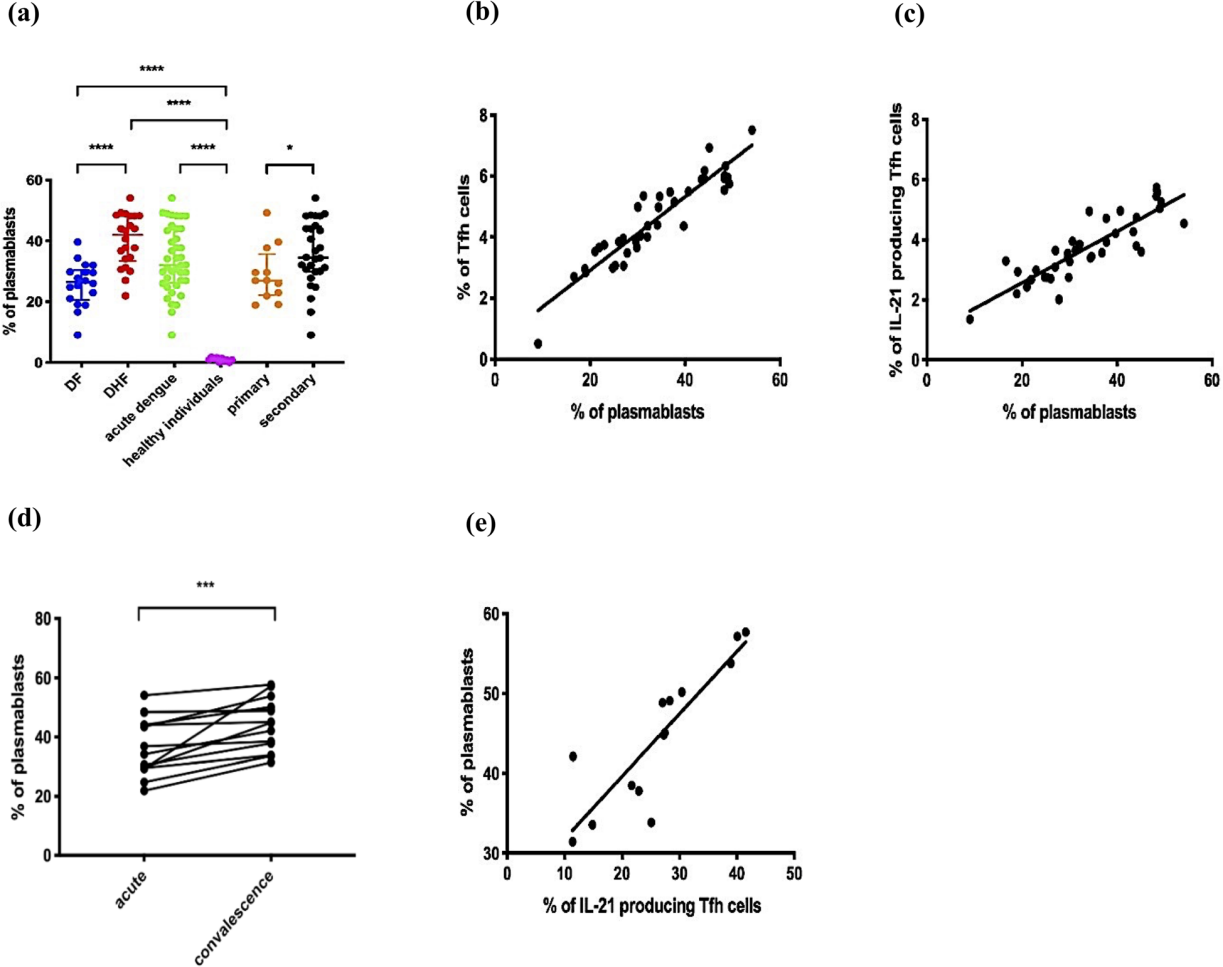
Plasmablast expansion in patients with varying severity of acute dengue infection and correlation of frequency of plasmablasts with frequency of Tfh cells. The frequency of plasmablasts was measured in patients with DF (*n* = 18), DHF (*n* = 22), primary dengue infection (*n* = 13), secondary infection (*n* = 27), acute DENV2 infection (*n* = 40) and healthy individuals (*n* = 12) (**a**). The frequency of plasmablasts (*n* = 14) was correlated with the frequency of Tfh cells in acute infection (Spearman *r* = 0.91, *p* < 0.0001) (**b**) and with the frequency of IL-21 producing Tfh cells in acute infection (Spearman *r* = 0.78, *p* < 0.0001) (**c**). The frequencies of plasmablasts were compared between individual patients between the acute and convalescent phase (**d**) and the plasmablast frequencies in the convalescent phase were correlated with the frequency of IL-21 producing Tfh cells during convalescent phase (Spearman *r* = 0.92, *p* < 0.0001) (**e**). The lines display the median and the IQR. *P < 0.05, ***p < 0.001, ****p < 0.0001

Since Tfh cells activate germinal centre B cells and Tfh cell cytokines such as IL-21 induce proliferation of plasmablasts, we investigated the association of the Tfh cell activation and cytokine production with the frequency of plasmablasts (Supplementary fig. 3). A representative flow cytometry plot for plasmablasts in a patient with acute infection and during convalescence is shown in supplementary figure 4. As shown previously [16, 22], we too found that plasmablasts significantly expanded (*p* < 0.0001) during acute infection compared to healthy individuals (Fig. 3a). The frequencies of plasmablasts associated with clinical disease severity were significantly higher (*p* < 0.0001) in patients with DHF when compared to those with DF (Fig. 3a). The frequency of plasmablasts was also significantly higher (*p* = 0.03) in patients with secondary dengue infections to those with primary infections (Fig. 3a).

The frequency of plasmablasts significantly and positively correlated with the frequency of the total Tfh cells (Spearman * r* = 0.91, *p* < 0.0001) (Fig. 3b). Although the frequency of plasmablasts correlated with Tfh cells expressing PD1 + ICOS+ (Spearman *r* = 0.37, *p* = 0.02) and with PD1 + ICOS- (Spearman *r* = 0.34, *p* = 0.03) the greatest correlation was observed with Tfh cells producing IL-21 (Spearman *r* = 0.78, *p* < 0.0001) (Fig. 3c).

As with the frequency of Tfh cells, we also determined the frequency of plasmablasts in the convalescent phase in 14/40 patients in this cohort. The frequency of plasmablasts was significantly higher (*p* = 0.0001) in convalescent than in the acute phase (Fig. 3d and Supplementary fig. 4). Similar to the observations in the acute phase of illness, the frequency of plasmablasts significantly correlated with the frequency of IL-21 secreting Tfh cells in the convalescent phase (Spearman *r* = 0.92, *p* < 0.0001) (Fig. 3e). In summary, these data show that expansion of plasmablasts was associated with clinical disease severity and with the frequency of Tfh cells, and that Tfh cells which had the highest functional capacity (those that produce IL-21) could be associated with an increase in plasmablasts.

### Association of Tfh cells with virological and laboratory parameters and DENV envelope protein specific antibody responses

**Fig. 4:**
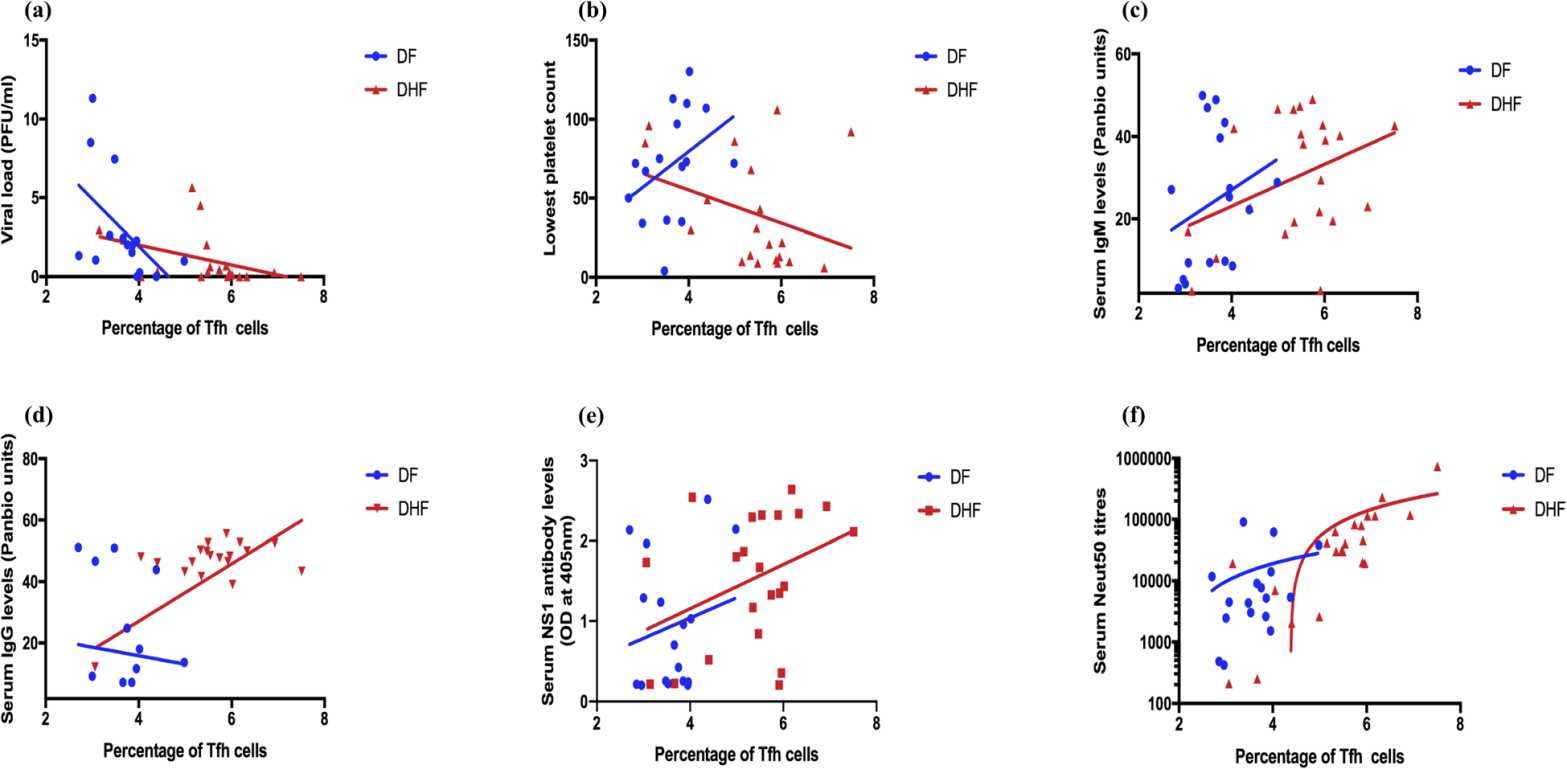
Association of follicular helper T cells with laboratory parameters and DENV-specific antibodies. The frequency of Tfh cells were measured in patients with DF indicated in blue (*n* = 18) and DHF indicated in red (*n* = 22) during the acute DENV2 infection and correlated with the viral loads (**a**), the lowest platelet counts (**b**), DENV-specific IgM antibodies (**c**), DENV-specific IgG antibodies (**d**), DENV-specific NS1 antibodies (**e**), and DENV2 specific neutralizing antibody titers (Neut50) (**f**)

The frequency of Tfh cells inversely correlated with the viral loads (Spearman *r* = − 0.63, *p* < 0.0001) of patients with acute dengue, suggesting that Tfh cell expansion was associated with resolution of viraemia. The association with viraemia and Tfh cell frequency was more significant in patients with DHF (Spearman *r* = − 0.59, *p* = 0.006), than in patients with DF (Spearman *r* = − 0.62, *p* = 0.01) (Fig. 4a). Although a significant inverse correlation was seen with the frequency of IL-21 producing Tfh cells and the viral loads (Spearman *r* = − 0.57, *p* = 0.0002), no correlation was observed between PD1 + ICOS+, PD1 + ICOS-, PD1-ICOS+ subsets of Tfh cells. The frequency of Tfh cells did not show any correlation with platelet counts in patients with either DF (Spearman *r* = 0.31, *p* = 0.2) or DHF (Spearman *r* = − 0.1, *p* = 0.9) (Fig. 4b).

In order to study the relationship between Tfh cells, plasmablasts and DENV specific antibodies we investigated the association of Neut50, total DENV antibodies and also DENV NS1 specific antibodies in this cohort of patients. We semi-quantitatively measured DENV-specific IgM and IgG antibody levels in serum (expressed as Panbio units), using the Panbio IgM and IgG capture ELISA, which uses the DENV envelope protein as the coating antigen. There was no significant difference (*p* = 0.42) in DENV-specific IgM titres between patients with DF and DHF, whereas DENV-specific IgG titres were significantly higher (*p* = 0.004) in DHF patients than in those with DF. We detected no significant correlation between DENV-specific IgM antibody titres and frequency of Tfh cells in patients with DF (Spearman *r* = 0.15, *p* = 0.54) and DHF (Spearman *r* = 0.25, *p* = 0.24) (Fig. 4c). The DENV-specific IgG titres also did not correlate with the frequency of Tfh cells in patients with DF (Spearman *r* = 0.08, *p* = 0.73), but a significant correlation was seen in patients with DHF (Spearman *r* = 0.43, *p* = 0.04) (Fig. 4d). Collectively, these data suggest that high levels of DENV-specific IgG levels seen in patients with DHF could associate with clinical disease severity.

### Association of plasmablasts with virological and laboratory parameters and DENV envelope protein specific antibody responses

**Fig. 5:**
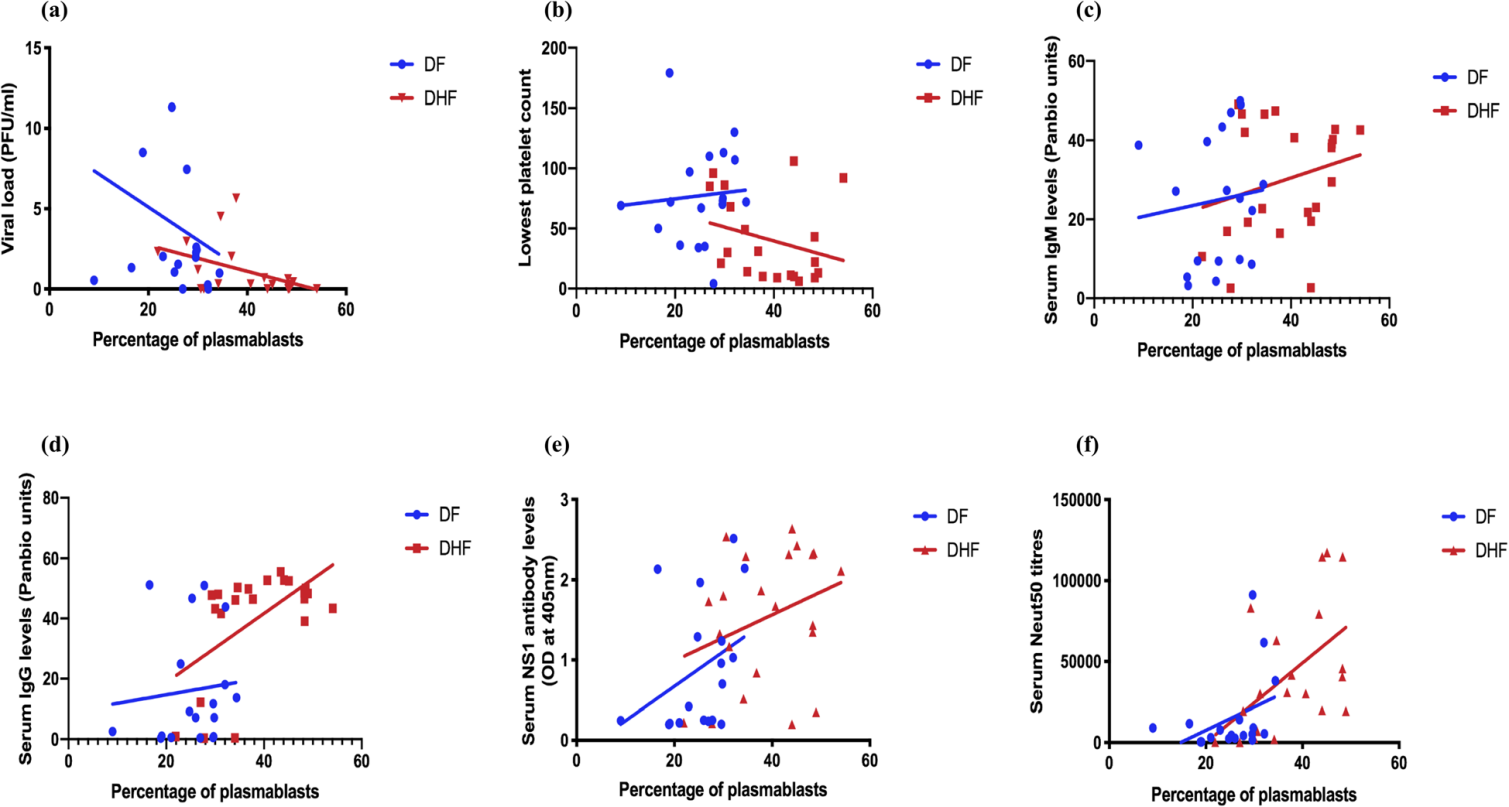
Association of plasmablasts with laboratory parameters and DENV-specific antibodies. The frequency of plasmablasts were measured in patients with DF indicated in blue (*n* = 18) and DHF indicated in red (*n* = 22) during the acute DENV2 infection and correlated with the viral loads (**a**), the lowest platelet counts (**b**), DENV-specific IgM antibodies (**c**), DENV-specific IgG antibodies (**d**), DENV-specific NS1 antibodies (**e**), and DENV2 specific neutralizing antibody titres (Neut50) (**f**)

As seen with Tfh cells, a significant inverse correlation was seen between the frequency of plasmablasts and the viral loads in patients with DHF (Spearman *r* = − 0.51, *p* = 0.02), but not in patients with DF (Spearman *r* = − 0.29, *p* = 0.26) (Fig. 5a). No association was seen with the frequency of plasmablasts and the lowest platelet count in the patients with DF (Spearman *r* = 0.33, *p* = 0.17) or DHF (Spearman *r* = − 0.35, *p* = 0.13) (Fig. 5b).

As observed with Tfh cells, DENV-specific IgM antibody titres did not correlate with the frequency of plasmablasts in patients with DF (Spearman *r* = 0.27, *p* = 0.26) or in DHF (Spearman *r* = 0.21, *p* = 0.34) (Fig. 5c). Similarly, the frequency of plasmablasts showed no significant correlation with DENV-specific IgG titres in DF patients (Spearman *r* = 0.18, *P* = 0.45) but significantly correlated with DENV-specific IgG in patients with DHF (Spearman *r* = 0.48, *p* = 0.02) (Fig. 5d). Therefore, as seen with the frequency of Tfh cells in patients with DHF, the frequency of plasmablasts appears to associate with DENV-specific IgG titres only in patients with DHF.

### Association of Tfh cells and plasmablasts with DENV-specific NS1 antibody responses

Although the majority of DENV-specific antibodies are specific to the DENV envelope protein, 27% of the DENV antibody repertoire has shown to be specific to DENV NS1 protein [35]. Therefore, we measured DENV2 specific NS1 antibodies using an in-house ELISA as described before [18]. As observed earlier, NS1 antibody levels were significantly higher in patients with DHF compared to those with DF. A significant correlation was not observed between NS1 antibody levels and frequencies of Tfh cells in patients with DF (Spearman *r* = 0.23, *p* = 0.35) and DHF (Spearman *r* = 0.32, *p* = 0.14) (Fig. 4e). Similarly, we did not observe a significant correlation between NS1 antibody levels and frequencies of plasmablasts in both DF (Spearman *r* = 0.44, *p* = 0.06) and DHF (Spearman *r* = 0.29, *p* = 0.18) (Fig. 5e). As shown in our earlier cohorts [18], the NS1 antibody levels inversely correlated with platelet counts (Spearman *r* = − 0.38, *p* = 0.02) and with viral loads (Spearman *r* = − 0.5, *p* = 0.001). Therefore, these data suggest that there was no association between the frequency of plasmablasts or Tfh cells with the NS1 antibody levels in patients with DF or DHF.

### Association of Tfh cells and plasmablasts with DENV- specific neutralizing antibody titres

**Fig. 6:**
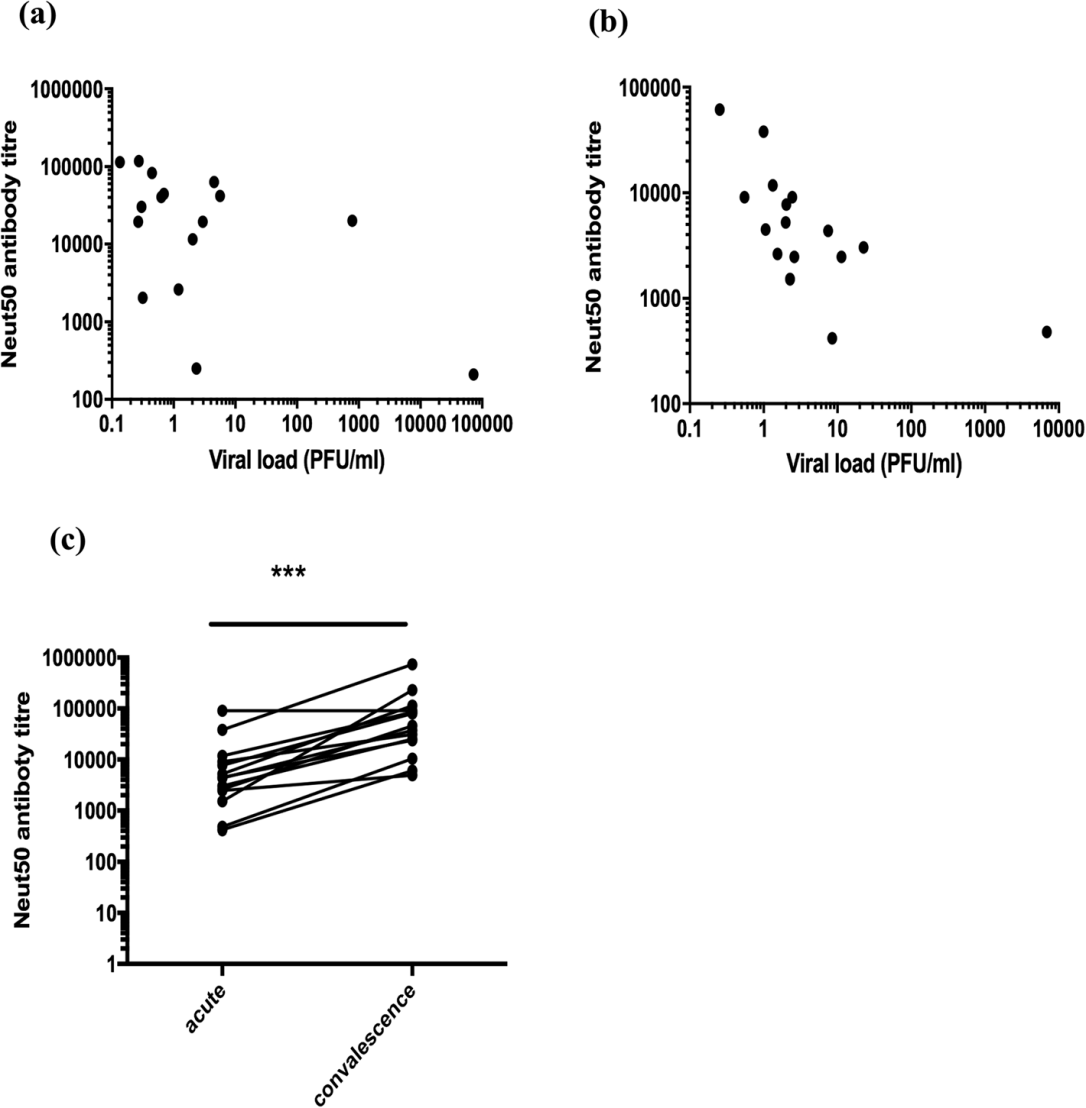
Association of neutralizing antibody titres (Neut50) with viraemia and changes with time. The Neut50 titres were measured in patients with DF (*n* = 18) and DHF (*n* = 22) during the acute DENV2 infection and correlated with the viral loads. A significant and negative correlation of viral loads with Neut50 antibody titres were seen in patients with DF (Spearman *r* = − 0.75, *p* = 0.003) (**a**) but not with DHF (Spearman *r* = − 0.26, *p* = 0.23) (**b**). Neut50 titres were also compared in patients during acute illness (day 4 to 8, *n* = 40) and during the convalescent phase (day 21 to 30, *n* = 14) (**c**). ***p < 0.001

A significant inverse correlation was seen in the viral loads with DENV-specific IgG titres (Spearman *r* = − 0.34, *P* = 0.03) and with DENV-specific NS1 antibody levels (Spearman *r* = − 0.48, *p* = 0.002). However, as the Panbio ELISA measured all DENV envelope specific antibodies and not necessarily neutralizing antibodies, which are thought in some studies to associate with protection, we then proceeded to determine association of neutralizing antibodies specific to the infecting DENV serotype (DENV2) with the frequency of Tfh cells and plasmablasts. The Neut50 titres were significantly higher (*p* = 0.006) in patients with DHF than in the patients with DF. Although there was no significant correlation in Neut50 titres with the frequency of Tfh cells in patients with DF (Spearman *r* = 0.30, *p* = 0.21), a significant correlation was detected between Neut50 titres and the frequency of Tfh cells in patients with DHF (Spearman *r* = 0.80, *p* < 0.0001) (Fig. 4f). We observed the same relationship with the frequency of plasmablasts. i.e. there was no significant correlation between Neut50 titres and the frequency of plasmablasts in patients with DF (Spearman *r* = 0.42, *p* = 0.08) but in patients with DHF (Spearman *r* = 0.61, *p* = 0.002) (Fig. 5f). Interestingly, in this cohort of patients who all had a DENV2 infection, Neut50 titres only inversely correlated with the viral loads in patients with DF (Spearman = − 0.75, *p* = 0.0003) and not in those with DHF (Spearman *r* = − 0.26, *p* = 0.23) (Fig. 6a and b, respectively).

The Neut50 titres were measured also in the convalescent phase (day 21 to 30 since onset of illness) in 14/40 patients with acute dengue (DF = 3, DHF = 11) of which 4 patients were with primary DENV2 infection and 10 patients with secondary DENV2 infection. The Neut50 titres were 10 fold higher (*p* = 0.0002) in the convalescent phase (median 41,432, IQR = 20,609 to 97,105 Neut50 titres) when compared to the acute phase (median 4416, IQR = 2220 to 9777 Neut50 titres) (Fig. 6c).

### Kinetics of neutralizing antibody production in patients with varying severity of acute DENV1 and DENV2 infection

The above data show that Neut50 antibody titres significantly correlated with the frequency of Tfh cells and plasmablasts in patients with DHF but not in those with DF and also inversely correlated with the viral loads only in patients with DF. Since the association of Neut50 titres with clinical disease severity has not been assessed, we sought to investigate the changes in the Neut50 titres throughout the course of illness in patients with an acute DENV1 (day 5–8 since onset of illness) or an acute DENV2 (day 6–9 since onset of illness) infection.

As the Neut50 titres are likely to be different in those with primary and secondary dengue longitudinally, in order to compare disease severity, we investigated the kinetics of Neut50 titres in a cohort of patients with secondary dengue (this is a different cohort than the patients recruited for the Tfh cell studies). We studied the kinetics of the Neut50 titres in 39 patients with varying severity of acute DENV1 infection (DF = 12, DHF = 10) and DENV2 infection (DF = 8, DHF = 9) throughout the course of illness. The first sample was obtained before any of the patients proceeded to develop either DF or DHF.

**Fig. 7:**
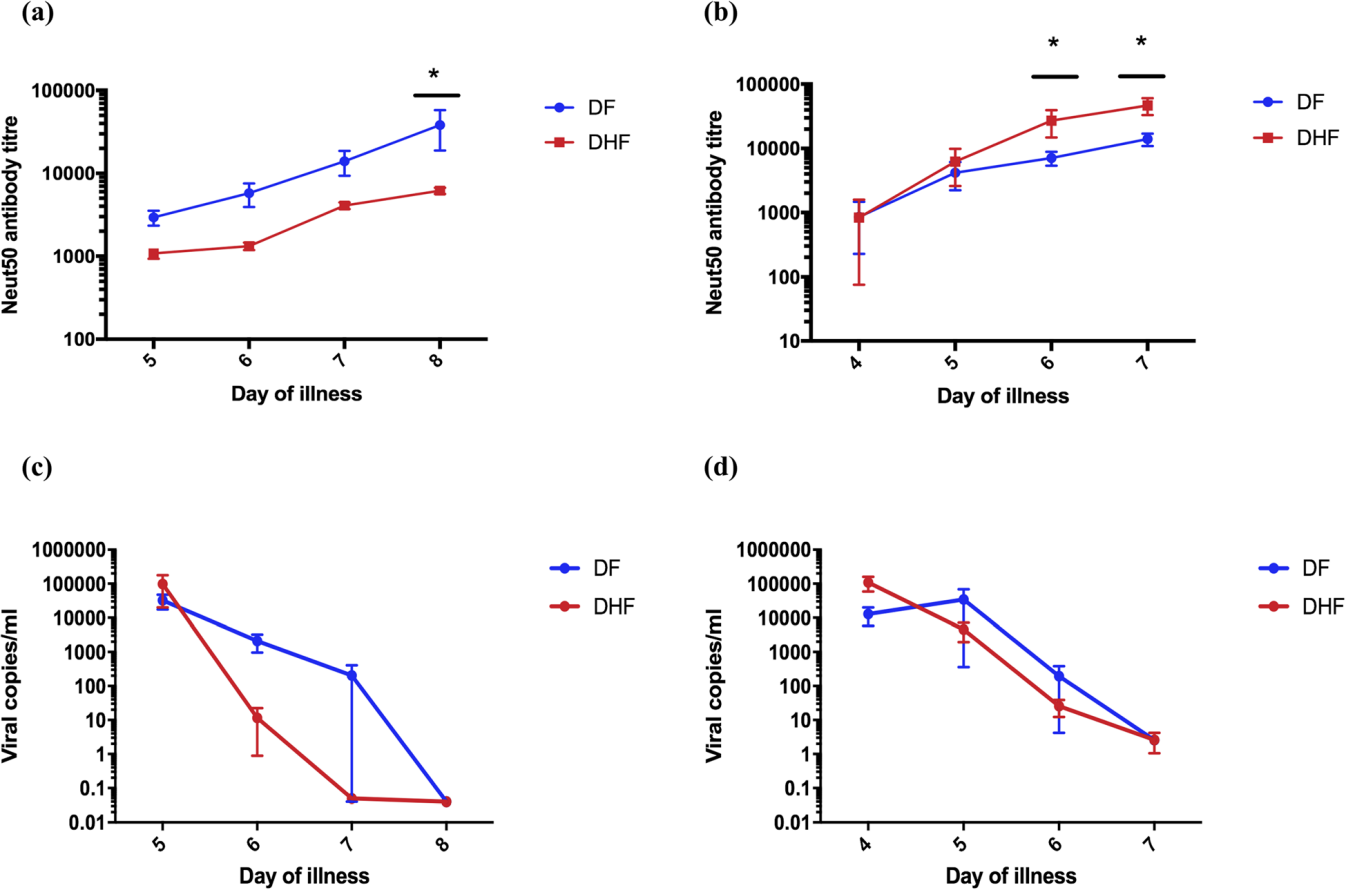
Kinetics and association of neutralizing antibody titres with viral loads. Neutralizing antibody (Neut50) titres were assessed in acute secondary DENV1 infection in patients with DF indicated in blue (*n* = 12) and DHF in red (*n* = 10) (**a**) and in acute secondary DENV2 infection in patients with DF indicated in blue (*n* = 8) and DHF in red (*n* = 9) (**b**) throughout the course of illness from date of admission to discharge. Viral loads were also measured in the same cohort of patients with acute secondary DENV1 infection in patients with DF (*n* = 12) and DHF (*n* = 10) (**c**) and in acute secondary DENV2 infection in patients with DF (*n* = 8) and DHF (*n* = 9) (**d**) throughout the course of illness from date of admission to discharge

We found that the Neut50 titres of those with acute secondary DF and DHF in both DENV1 and DENV2 infections were similar in the febrile phase before the onset of the plasma leakage in patients with DHF (Fig. 7a and b). While the Neut50 titres exponentially increased in patients with DF in acute secondary DENV1 infection, the opposite was observed in acute secondary DENV2 infection. However, there was no significant difference in the viral loads in patients with DF and DHF at any time point in the illness in acute secondary DENV1 infection (Fig. 7c) or in acute secondary DENV2 infection (Fig. 7d). The viral loads at the beginning of the illness did not correlate with the Neut50 titres seen in the course of illness in patients with DF or DHF in DENV1 infection at any time point measured. No such associations were also observed for either DF or DHF in acute secondary DENV2 infection.

At the end of the critical phase (in the recovery phase) the Neut50 antibody titres were several folds higher in those with both DF and DHF when infected with DENV2 compared to DENV1. For instance, those with an acute secondary DENV2 causing DHF had a median Neut50 titre of 43,652 (IQR = 29,512 to 69,183) compared to those with DF (median 14,234, IQR = 8913 to 19,055). In contrast, those with an acute secondary DENV1 causing DHF had a median Neut50 titre of 5890 (IQR = 4266 to 8913) compared to those with DF (median 17,819, IQR = 7943 to 251, 188).

## Discussion

Our results show that Tfh cells are significantly expanded in acute dengue, especially in those with severe clinical disease (DHF). There was also a significant increase in the IL-21 producing Tfh cells in patients with DHF, which are the most functional subset of Tfh cells and therefore, most capable of providing help to B cells [14]. The Tfh cells co-expressing PD-1 + ICOS+, which are the subtype of Tfh cells that are most efficient in inducing germinal centre B cell antibody production [15], were also significantly expanded, especially in those with DHF. Although all types of Tfh cells (PD-1 + ICOS+, PD-1 + ICOS-, PD1-ICOS+) correlated with the frequency of the plasmablasts, the IL-21 producing Tfh cells showed the most significant and the strongest correlation, suggesting that the most functional subset of Tfh cells was driving the plasmablast expansion in acute dengue infection. However, although both PD-1 and ICOS co-expressing Tfh cells were expanded in acute illness, the majority of Tfh cells only expressed PD-1. Since PD-1 expression by Tfh cells has been shown to limit the Tfh cell recruitment to the germinal centre, limit T cell receptor signalling and confine these cells to the germinal centre [34, 36], they could have a regulatory role by balancing the B cell activation by IL-21 and PD-1, ICOS co-expressing Tfh cells.

Although the Tfh cells in general and the IL-21 producing Tfh cells further expanded in the convalescent phase along with plasmablasts, PD-1 expressing Tfh cells significantly decreased suggesting that although the Tfh cells were functional, they were less activated during the convalescent phase. Production of neutralizing antibodies appear to continue after the acute phase (day 6 to 8 measured in this study) as the Neut50 titres were 10 times higher in the convalescent phase compared to the values in the acute phase. The Neut50 titres in the convalescent phase only correlated with the IL-21 producing Tfh cells, suggesting that this functional subset of Tfh cells is likely to be important to drive high titres of long-lasting neutralizing antibody titres.

The total population of Tfh cells and plasmablasts significantly correlated with Neut50 antibody titres and DENV-specific IgG antibodies in patients with DHF, indicating their importance in driving pathogen specific antibody responses during acute infection. Therefore, the higher DENV-specific IgG, Neut50 titres and NS1-antibody levels seen in patients with DHF compared to those with DF, is probably due to increased numbers of plasmablasts producing higher levels of DENV-specific antibodies. The frequency of Tfh cells, plasmablasts, DENV specific IgG and Neut50 titres all inversely correlated with the viral loads, which suggest that they are likely to be important in the control of the virus. The Neut50 antibody titres were significantly higher in patients with DHF, when assessed in a single time point, in the first cohort of patients infected with DENV2. This could be due to the presence of higher viral loads in patients with DHF that lead to increased expansion of Tfh cells and thus plasmablasts, resulting in higher Neut50 titres. However, when we measured the viral loads serially throughout the course of illness in patients with acute secondary DENV1 and DENV2 infection, we did not observe higher viral loads in patients with DHF compared to those with DF. However, since the viral loads were assessed from day 3 of illness onwards, there is a possibility that those with DHF had higher viral loads very early in illness. Such high viral loads in early illness that was not captured by us could have led to higher Tfh cell responses in patients with DHF.

During the febrile phase of illness, again no difference was seen in the infecting viral specific Neut50 titres in patients with either DENV1 or DENV2 infection who proceeded to develop either DF or DHF. Therefore, the Neut50 titres at the beginning of illness, does not appear to associate with subsequent clinical disease severity. Interestingly, in DENV1 infection the Neut50 antibody titres significantly rose in those with DF compared to those with DHF throughout the course of illness, whereas the opposite was seen in DENV2 infection (Neut50 titres rose higher in DHF > DF). This rise in the Neut50 titres throughout the illness was not associated with the viral loads seen in early illness in either DF or DHF patients with DENV1 and DENV2. Therefore, these data show that the degree of viraemia during early illness was not associated with the degree of subsequent rise in Neut50 titres. In addition, the rise in neutralizing antibody titres does not appear to associate with clinical disease severity but appears to be very different between DENV1 and DENV2. Previous studies have also shown that the degree and duration of viraemia varied between DENV serotypes and there were no differences in the degree of viraemia and kinetics between patients with DF and DHF [37–39]. Collectively, these data show that the rise in the Neut50 titres did not depend on the extent of viraemia; Neut50 titres in the febrile phases in both acute secondary DENV1 and DENV2 were similar in patients who subsequently progressed to develop either DF or DHF.

Although a Neut50 antibody titre of 1:10 is considered protective in some studies [27, 40], some individuals with high neutralizing antibodies for a particular DENV serotype later went on to develop DHF when infected with that particular serotype [41]. Furthermore, those who were found to have high Neut50 titres to DENV2 in phase 2b and 3 dengue vaccine trials, were later found to be infected with the same serotype when naturally exposed to the virus [42, 43]. In this study too, patients who later progressed to develop DHF had similar Neut50 antibody titres as patients who developed DF. Indeed, in a dengue vaccine trial, individuals with high neutralizing antibody titres to certain DENV serotypes were not protected with infection with those serotypes, questioning the Neut50 antibody titres that associate with protection [9, 42]. Therefore, Neut50 antibody titres during the febrile phase measured by in vitro assays do not appear to reflect a good correlate of in vivo protection.

## Conclusions

In summary, the Tfh cells appear to significantly expand in acute dengue, which associated with clinical disease severity and plasmablast expansion. This increase in frequency also correlated with DENV-specific IgG, Neut50 titres and with NS1-specific antibody titres. Since evaluation of Neut50 titres in the febrile phase (before patients developed either DF or DHF) did not appear to associate with clinical disease severity or the degree of viraemia, neutralizing titres alone do not appear to determine protection against severe clinical disease.

## Supplementary information

**Additional file 1: Supplementary figure 1.** The hierarchical gating strategy used to identify Tfh cells and to assess their phenotype and functionality. **Supplementary figure 2.** The frequency of Tfh cells and IL-21 producing Tfh cells in patients with acute dengue during acute phase and convalescent phase. **Supplementary figure 3.** The hierarchical gating strategy used to identify plasmablasts. Cells were first gated on the PBMCS, then the singlets were identified by gating on FSC-height and area. These cells were then gated on the live cells and subsequently on CD19, CD27 and CD38. **Supplementary figure 4.** The frequency of plasmablasts in a patient with acute dengue in the acute and convalescent phase.

**Additional file 2: Supplementary table 1.** Clinical and laboratory characteristics of patients with DHF and DF. **Supplementary table 2.** Definitions of terms with regard to DENV infections.

## Data Availability

All data generated or analyzed during this study are included in this published article and its supplementary information files.
