# Supplementary material for: Phenotype and functionality of follicular helper T cells in patients with acute dengue infection
Source: J Biomed Sci. 2020 Apr 8;27:50. doi: 10.1186/s12929-020-00641-2 (PMC7140349; doi:10.1186/s12929-020-00641-2)
Supplement: Supplementary file 1 — Additional file 1: Supplementary figure 1. The hierarchical gating strategy used to identify Tfh cells and to assess their phenotype and functionality. Supplementary figure 2. The frequency of Tfh cells and IL-21 producing Tfh cells in patients with acute dengue during acute phase and convalescent phase. Supplementary figure 3. The hierarchical gating strategy used to identify plasmablasts. Cells were first gated on the PBMCS, then the singlets were identified by gating on FSC-height and area. These cells were then gated on the live cells and subsequently on CD19, CD27 and CD38. Supplementary figure 4. The frequency of plasmablasts in a patient with acute dengue in the acute and convalescent phase. [file 12929_2020_641_MOESM1_ESM.docx]

**Supporting information data**


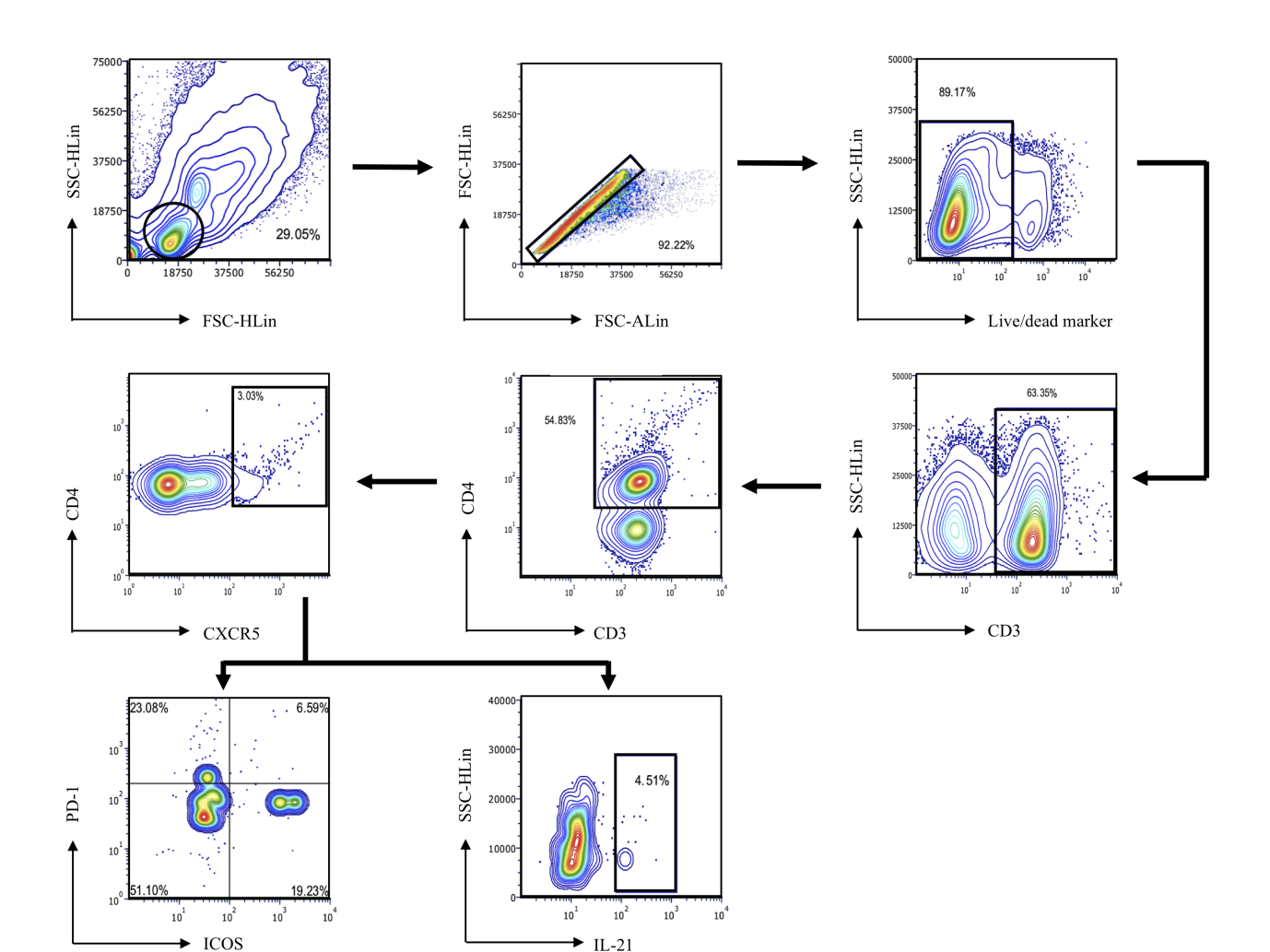


**Supplementary figure 1: The hierarchical gating strategy used to identify Tfh cells and to assess their phenotype and functionality.** Cells were first gated on the PBMCS, then the singlets were identified by gating on FSC-height and area. These cells were then gated on the live cells and subsequently on CD3+ cells. The Tfh cells were identified as those expressing CD4 and CXCR5. These Tfh cells were subsequently gated on PD-1, ICOS and IL-21 to determine their expression levels.


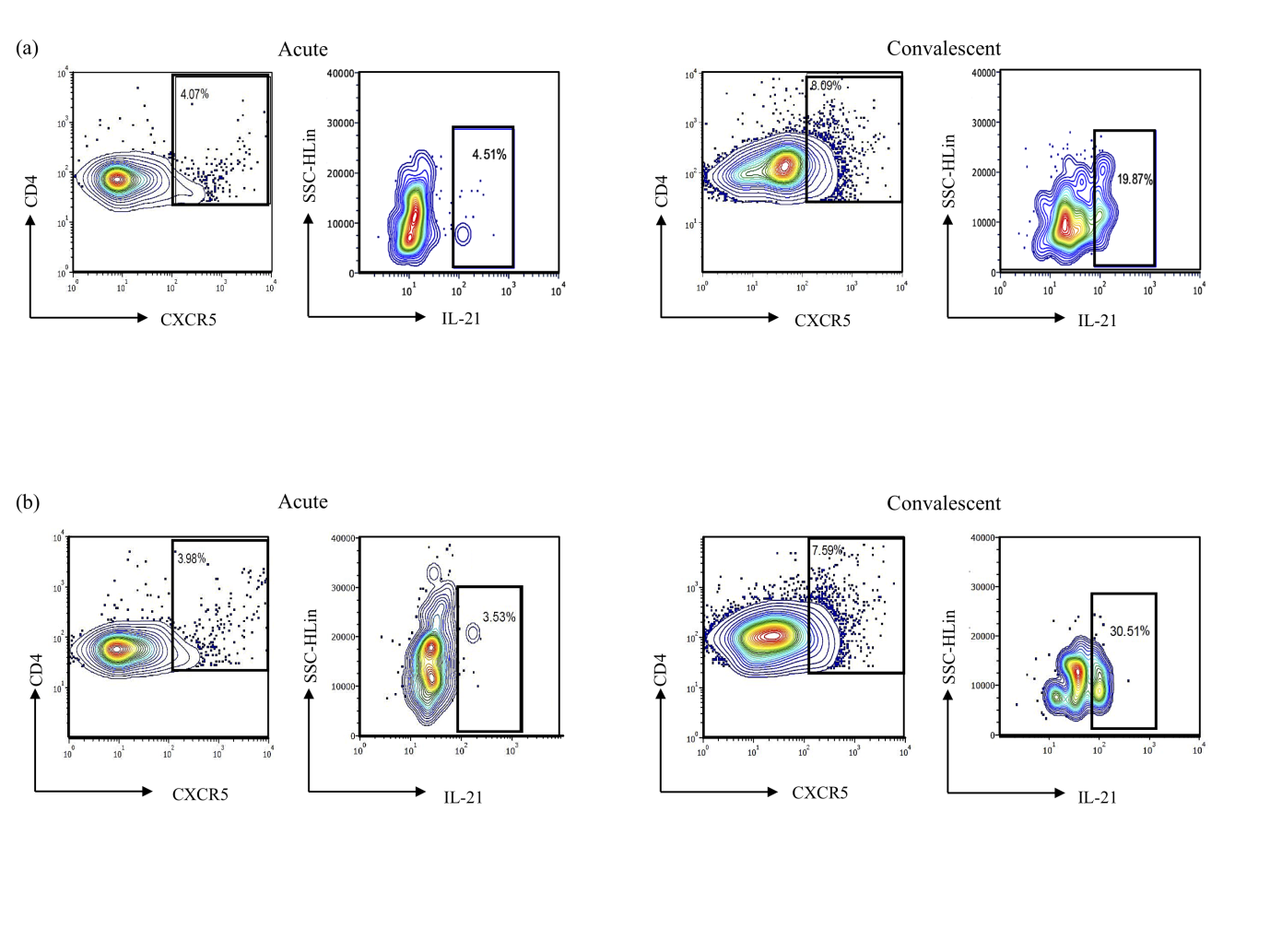


**Supplementary figure 2: The frequency of Tfh cells and IL-21 producing Tfh cells in patients with acute dengue during acute phase and convalescent phase**

The expression of total Tfh cells (cells expressing CD4+CXCR5+) and IL-21 producing Tfh cells in two patients; patient (a) during acute (day 7 since onset of illness) and convalescent phases (day 25 since onset of illness) and patient (b) during acute (day 6 since onset of illness) and convalescent phases (day 25 since onset of illness) are shown.


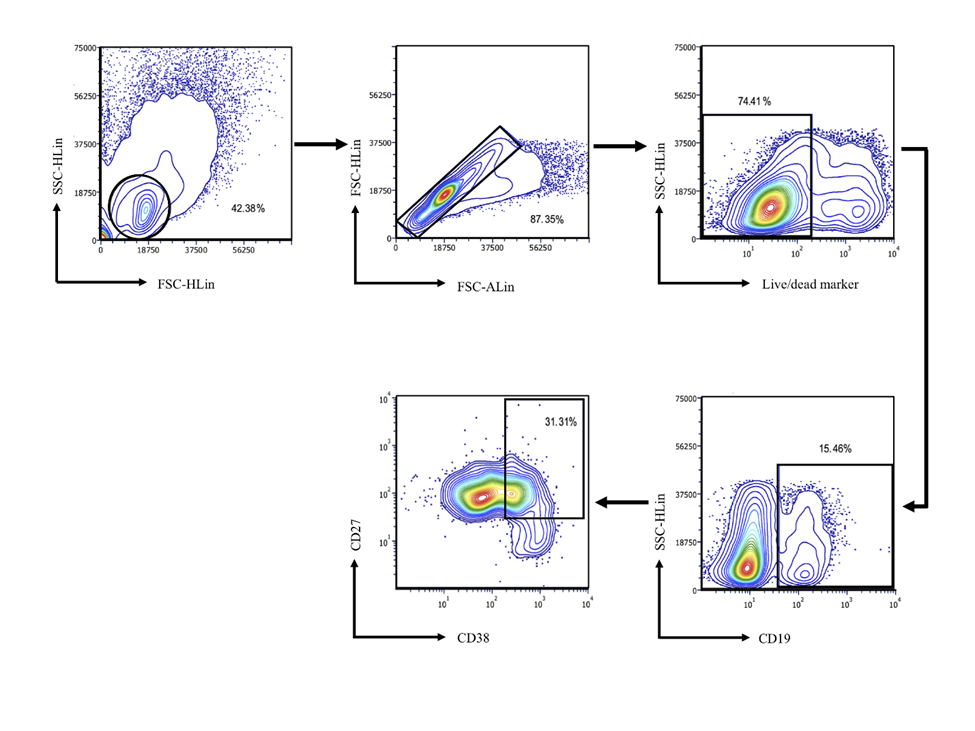


**Supplementary figure 3: The hierarchical gating strategy used to identify plasmablasts.** Cells were first gated on the PBMCS, then the singlets were identified by gating on FSC-height and area. These cells were then gated on the live cells and subsequently on CD19, CD27 and CD38.


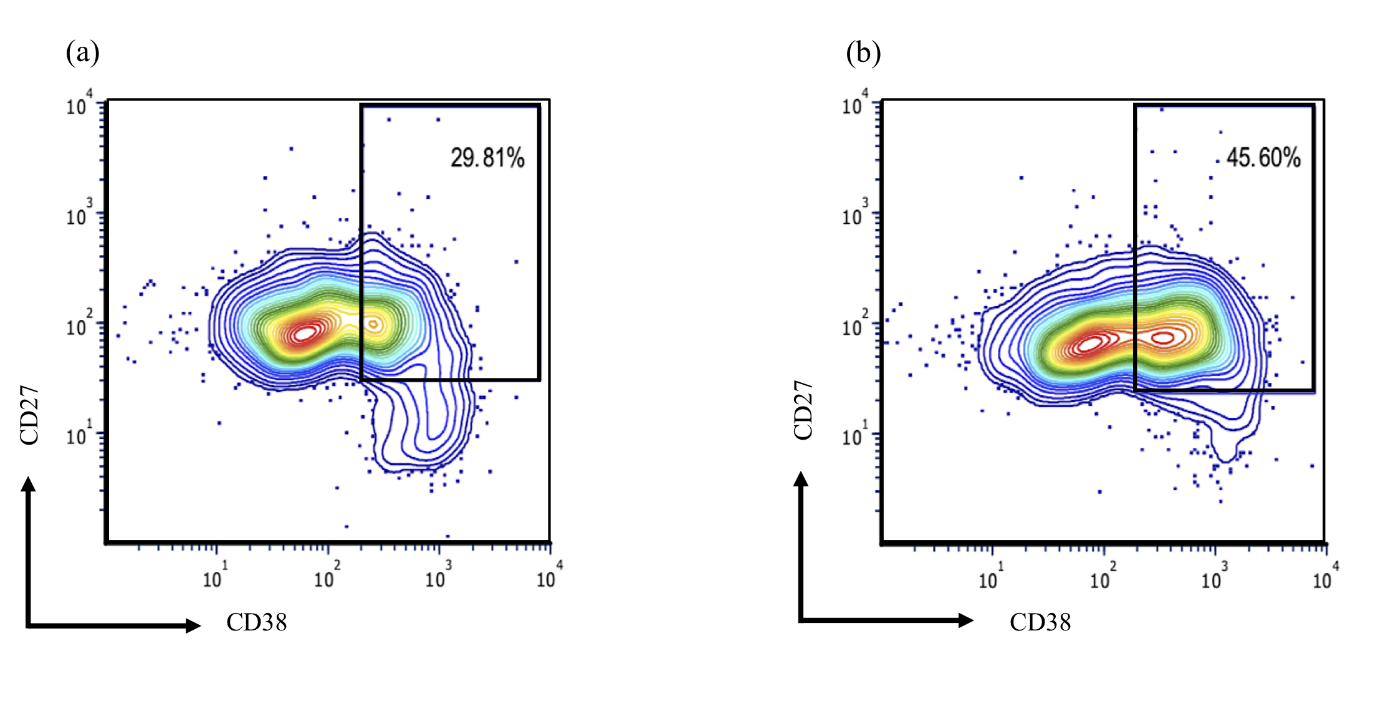
 **Supplementary figure 4: The frequency of plasmablasts in a patient with acute dengue in the acute and convalescent phase**

The expression of plasmablasts (CD19 cells expressing CD38 and CD27) are shown in the same patient during the acute (a) and convalescent phase (b).
