# Supplementary material for: Phenotype and functionality of follicular helper T cells in patients with acute dengue infection
Source: J Biomed Sci. 2020 Apr 8;27:50. doi: 10.1186/s12929-020-00641-2 (PMC7140349; doi:10.1186/s12929-020-00641-2)
Supplement: Supplementary file 2 — Additional file 2: Supplementary table 1. Clinical and laboratory characteristics of patients with DHF and DF. Supplementary table 2. Definitions of terms with regard to DENV infections. [file 12929_2020_641_MOESM2_ESM.docx]

**Supplementary table 1: Clinical and laboratory characteristics of patients with DHF and DF**

| Clinical findings | DHF  N=22 | DF  N=18 |
| --- | --- | --- |
| Vomiting | 7 (31.8%) | 1 (5.6%) |
| Abdominal pain | 12 (54.6%) | 3 (16.7%) |
| Hepatomegaly | 6 (27.3%) | 1 (5.6%) |
| Bleeding manifestations | 2 (9.1%) | 0 (0%) |
| Pleural effusion | 5 (22.7%) | 0 (0%) |
| Ascites | 21 (95.5%) | 0 (0%) |
| Lowest platelet counts (cells/ mm^3^) | | |
| <20,000 | 10 (45.5%) | 1 (5.6%) |
| 20,000 to 50,000 | 6 (27.3%) | 4 (22.2%) |
| 50,000–100,000 | 5 (22.7%) | 8 (44.4%) |
| >100,000 | 1 (4.5%) | 5 (27.8%) |
| Lowest Lymphocyte counts (cells/ mm^3^) | | |
| <750 | 10 (45.5%) | 4 (22.2%) |
| 750–1500 | 10 (45.5%) | 13 (72.2%) |
| >1500 | 2 (9.1%) | 1 (5.6%) |

**Supplementary table 2: Definitions of terms with regard to DENV infections**

| Term | Definition |
| --- | --- |
| Acute infection | A patient who has an acute illness due to infection with a dengue virus. All those who were included in this study and defined as having acute infection, were acutely ill and hospitalized due to dengue infection |
| Convalescence | The patients who were included in the convalescent phase were considered to be fully recovered and samples obtained from day 21 to 30 from the day of onset of fever due to dengue |
| Primary infection | The initial infection of a host by a particular serotype of DENV, which is reflected by the DENV specific IgM: IgG ratio of >1.2 as per 2011 WHO guidelines |
| Secondary infection | Subsequent infection with the same or a different serotype of DENV, which is reflected by the DENV specific IgM: IgG ratio of <1.2 as per 2011 WHO guidelines |
